# Pathogenicity of *Arcobacter cryaerophilus* in two human intestinal cell lines

**DOI:** 10.1186/s13099-025-00721-4

**Published:** 2025-06-22

**Authors:** Antonia Bachus, Sarah Beyer, Roland Bücker, Soroush Sharbati, Thomas Alter, Greta Gölz

**Affiliations:** 1 Institute of Food Safety and Food Hygiene, Freie Universität Berlin, Berlin, Germany; 2 Clinical Physiology/Nutritional Medicine, Charité– Universitätsmedizin Berlin, Berlin, Germany; 3 Institute of Veterinary Biochemistry, Freie Universität Berlin, Berlin, Germany

**Keywords:** *Arcobacter cryaerophilus*, Cell culture, Pathogenicity, Cytotoxicity, Adhesion, Invasion, Epithelial barrier function

## Abstract

**Background:**

*Arcobacter cryaerophilus* is considered an emerging foodborne pathogen and is associated primarily with infectious gastrointestinal disease in humans. However, the underlying pathogenic mechanisms remain poorly understood. Therefore, the aim of the present study was to investigate the pathogenic potential of twelve *A. cryaerophilus* strains using various in vitro assays in two human colonic cell lines, HT-29/B6 and T84.

**Results:**

All strains tested were able to adhere to and invade into both cell lines, with strain-dependent differences in their adhesion and invasion rates. In addition, two strains showed cytotoxic effects on both cell lines. The ability to disrupt the epithelial barrier function of T84 cell monolayers was shown for two strains by measurement of transepithelial electrical resistance. As structural factors correlate with the barrier dysfunction, immunofluorescence staining of the tight junction domain was performed, and revealed an altered distribution of claudin-5 in infected cells.

**Conclusions:**

The results highlight the strain-dependent pathogenic mechanisms of *A. cryaerophilus* that may contribute to key symptoms such as diarrhoea. These findings also highlight the importance of further research into the pathogen *A. cryaerophilus*.

## Background

*Arcobacter* spp. are gram-negative rod-shaped bacteria belonging to the family of *Arcobacteraceae* [1–3]. To date, more than 30 species have been described, which are considered to be aerotolerant [4, 5].

Owing to their ability to adapt to different growth conditions, *Arcobacter* spp. have been isolated from a wide variety of different sources [6, 7]. These sources include environmental water, vegetables, seafood and various types of meat [8–16]. Additionally, both homothermic and poikilothermic animals were tested positive for *Arcobacter* spp. The majority of *Arcobacter* isolates were obtained from healthy animals, suggesting that *Arcobacteraceae* may act as a commensal in animals. However, few cases of *Arcobacter* isolation from aborted foetuses and cases of mastitis have been reported [17–21]. Moreover, numerous human stool samples were tested positive for *Arcobacter* spp. [22–24]. According to a prevalence study in Germany, *Arcobacter* spp. could be detected in 0.77% of tested human stool samples, whereby *Arcobacter* (*A.*) *butzleri* was the most frequently detected species (67%), and *A. cryaerophilus* represented the second most frequently detected species (28%) [25]. Similar results were also obtained in a survey in Belgium, with *Arcobacter* spp. being the fourth most frequent pathogen in human stool samples (1.3% in a 5-year survey). Consistent with the findings of Brückner et al., *A. cryaerophilus* was identified as the second most prevalent *Arcobacter* species (43%) following *A. butzleri* (55%) in faecal samples from humans with symptoms of enteritis [26]. Nevertheless, Houf et al. (2007) examined stool samples from healthy slaughterhouse staff and found 1.4% positive for *A. cryaerophilus*. No other *Arcobacter* species was detected in this study [27].

In addition, *Arcobacter* spp. are known to cause human disease, particularly gastrointestinal symptoms such as acute or prolonged watery diarrhoea, abdominal pain and nausea, but also more severe cases of bacteraemia have been reported [28–36]. Most of the case reports refer to *A. butzleri* and *A. cryaerophilus* as the causative pathogens [26]. Sporadic case reports also describe the detection of *A. skirowii*, *A. thereiu*s, and *A. mytili* in diseased humans [26, 37, 38]. Even though *A. lanthieri* has also been detected in human stool samples, no conclusion on its pathogenic potential could be drawn so far [39].

Owing to its harmful role in human health, *A. cryaerophilus* is classified as an emerging foodborne pathogen [40]. Despite this classification and in contrast to related species causing gastrointestinal illnesses in humans, such as *A. butzleri*, little is known about the clinical significance. Furthermore, the underlying pathogenic mechanisms of *A. cryaerophilus* remain poorly understood. Previous research on *A. cryaerophilus* has mainly focused on prevalence, antibiotic resistance, and growth characteristics, whereas only a limited number of studies have investigated pathogenic mechanisms, such as adhesion, invasion, and cytotoxicity for this species [4, 41–43]. These former studies consistently reported that *A. cryaerophilus* strains are capable of adhering and invading human intestinal cells, with some also observing cytotoxic effects [44–46]. However, due to the use of different cell lines, strains and protocols, it has not yet been possible to draw a connection between individual pathogenicity mechanisms. Moreover, the potential of *A. cryaerophilus* to affect the intestinal barrier function has not yet been investigated, although this might be of importance, given that diarrhoea is the most frequently reported symptom of *Arcobacter* spp. infections [29, 47, 48].

Therefore, this study aimed to investigate the pathogenic potential of *A. cryaerophilus* in two human intestinal cell lines, HT-29/B6 and T84. Adhesive, invasive and cytotoxic potentials were assessed using in vitro assays. Furthermore, the impact of *A. cryaerophilus* on the epithelial barrier was investigated by the measurement of the transepithelial electrical resistance and immunohistological staining of tight junction proteins. Our results provide a comprehensive in vitro analysis on the pathogenic properties of *A. cryaerophilus* and deeper insights into the mechanisms contributing to human diseases.

## Methods

### Bacterial strains and growth conditions

Twelve *A. cryaerophilus* strains isolated from several matrices, and without associated information on human clinical outcomes, were included in our study (Table 1). *A. butzleri* CCUG 30485 was included as control strain, except for immunofluorescence assay. All strains were stored in cryotubes (Mast Diagnostica, Reinfeld, Germany) at -80 °C. Unless otherwise stated, all bacterial cultures were cultivated under microaerobic conditions (5% O_2_, 10% CO_2_) generated by Anoxomat (AN2CTS, MART Microbiology, B.V., Drachten, Netherlands). All *Arcobacter* strains were cultivated at 30 °C.

**Table 1:** Strains included in the study

Species	Strain	Source	Reference
*A. cryaerophilus*	2486	Shrimp	[89]
	2502	Shrimp	[89]
	2517	Mussel	[89]
	2657	human stool sample	[45]
	2661	human stool sample	[45]
	2695	human stool sample	[25]
	2771	human stool sample	[45]
	2852	chicken meat	[22]
	3132	surface water	[22]
	3136	ready-to-eat salad	[22]
	3224	surface water	This study
	3226	surface water	This study
*A. butzleri*	30485	human stool sample, Culture Collection University of Gothenburg (CCUG)	https://www.ccug.se/

After the bacterial strains were streaked on Mueller-Hinton agar (Oxoid, Hampshire, UK) and incubated for 72 h, colonies were used to prepare precultures by inoculating Brucella Broth (Becton, Dickinson and Company, MD, USA) overnight. The precultures were diluted 1:100 in 10 mL of Brucella Broth and further incubated for 48 h (*A. cryaerophilus*) or 24 h (*A. butzleri*) to a final concentration of approximately 10^9^ CFU/mL. Subsequently, the achieved working cultures were centrifuged at the maximum speed of 7197 *× g* for 5 min, and the bacterial pellet was resuspended in 450 µL of the corresponding cell culture medium before infection of the host cells.

### Cell culture and growth conditions

The in vitro assays were performed on the human colon cell lines HT-29/B6 and T84. Both cell lines are of human colonic origin, whereby HT-29/B6 is a subclone of the cell line HT-29 that was derived from a primary colon adenocarcinoma and T84 was obtained from a lung metastasis of a colon adenocarcinoma [49, 50]. Cells were grown in 75 cm^2^ cell culture flasks in RPMI 1640 or Dulbecco´s Modified Eagle´s Medium/Ham´s Nutrient Mixture F-12 (DMEM/F12) (both Thermo Fisher Scientific, Waltham, MA, USA) supplemented with 10% foetal calf serum superior (Sigma-Aldrich, Darmstadt, Germany) and 10 µg/mL gentamicin (Sigma-Aldrich, St. Louis, MO, USA). Culturing conditions were 37 °C in a 5% CO_2_ humidified atmosphere. The medium was changed every second or third day.

### Cytotoxicity assay

Potential cytotoxic effects of *A. cryaerophilus* on human intestinal cell lines were tested using the WST-1 colorimetric assay as described by Brückner et al. [45]. Briefly, the cell-permeable WST-1 (4-[3-(4-iodophenyl)-2-(4-nitro-phenyl)-2 H-5-tetrazolio]-1,3-benzene sulfonate) can be cleaved by mitochondrial dehydrogenases of metabolically active cells to the orange dye formazan. Spectrophotometric measurements can be used to determine the amount of formazan formed, which is proportional to the metabolic activity of the cells. The absorbance measured from cells incubated with medium only (negative control) represents 100% viable cells. In a 96-well untreated, flat-bottomed polystyrene plate (Sarstedt, Nümbrecht, Germany), 3 × 10^4^ cells per well were seeded in 100 µL medium and differentiated for seven days at 37 °C and 5% CO_2_ humidified atmosphere until they formed confluent monolayers. After the cells were washed once with phosphate-buffered saline (PBS) (Carl Roth, Karlsruhe, Germany), 100 µL of fresh antibiotic-free medium was added. Subsequently, 50 µL of bacterial suspension (multiplicity of infection [MOI] = 1000) was added to each well and incubated for two days at 37 °C in a humidified 5% CO_2_ atmosphere. Afterwards, the cells were washed once with PBS and incubated for one hour with 100 µL medium supplemented with 10 µL of WST-1 solution (Merck, Darmstadt, Germany). Each supernatant was transferred to a fresh 96-well plate, and the absorbance was measured at 450 nm with a Clariostar Plus microplate reader (BMG Labtech, Ortenberg, Germany). The results were normalised to those of the negative control (medium). As a chemical positive control for cytotoxicity, 33% dimethyl sulfoxide (DMSO) (Carl Roth) was used. The WST-1 assay was performed for each strain in six technical replicates and was repeated in three independent experiments.

### Bacterial adhesion and invasion assay

A total of 1 × 10^5^ HT-29/B6 cells and 3 × 10^5^ T84 cells per well were seeded in 24-well untreated, flat-bottomed polystyrene plates (Sarstedt) and differentiated for seven days as described above until a confluent monolayer was achieved. One day prior to infection, the cells were incubated with antibiotic-free medium. Immediately before infection, the cells were washed once with PBS, and 500 µL of fresh antibiotic-free medium was added to each well. Then, 50 µL of bacterial suspension (MOI = 100) was added to each well, and the cells were incubated at 37 °C and 5% CO_2_. For the adhesion assay, infected cells were incubated for one hour to allow the bacteria to adhere to the cells. Afterwards, the cells were washed three times with PBS to remove the unbound bacteria and lysed with 500 µL of 1% Triton-X-100 (Sigma-Aldrich, St. Louis, MO, USA). The supernatant of each well was collected to determine the amount of adhered bacteria in the lysate by plating serial dilutions on Mueller-Hinton agar and incubating for 48 h. For the detection of invaded bacteria, a gentamicin protection assay was performed as described by Karadas et al. [51]. Briefly, 3 h post infection, the host cells were washed three times with PBS and incubated with media containing 300 µg/mL gentamicin for further two hours to ensure the killing of all extracellular bacteria. The cells were then washed three more times with PBS and lysed with 500 µL of 1% Triton-X-100. The amount of invaded bacteria was determined as described above. The ratios of adhered and invaded bacteria were calculated and compared with those of the inoculum. As a negative control, only medium was added.

All assays were performed in technical triplicates and were repeated in three independent experiments.

### Epithelial barrier function

The ability of five *A. cryaerophilus* strains to decrease the integrity of a confluently grown monolayer of polarised T84 cells was determined by measuring the transepithelial electrical resistance (TER) in Ω cm^− 2^.

For this purpose, 1 × 10^6^ cells were seeded on transwell filters (pore size 3 μm, diameter 12 mm; Merck Millipore, Billerica, MA, USA) and grown for two weeks until confluence, as described above. The TER between the apical and basal parts of the cell monolayer was measured via a Volt-Ohm meter and chopstick electrodes (EVOM3, World Precision Instruments, Sarasota, FL, USA). Following the measurement of the initial resistance, four different MOIs (10, 50, 100, and 500) of *A. cryaerophilus* strain 3136 were added apically to each of four transwell filters, and the cell culture was incubated at 37 °C and 5% CO_2_. The TER was remeasured 48 h post infection, and the results were normalised to the initial TER. This assay was performed in 4 replicates. In accordance, bacterial suspensions of four further *A. cryaerophilus* strains were tested for their ability to decrease the TER of T84 cell monolayers with a MOI of 100. *A. butzleri* CCUG 30485 was included as a positive control. The TER-assays of five different *A. cryaerophilus* strains were performed in technical triplicates and were repeated in three independent experiments.

### Immunofluorescence staining of tight junction proteins and confocal microscopy

T84 monolayers, grown on transwell filter supports and infected with *A. cryaerophilus* strain 3136 (MOI 100) for 48 h were fixed after TER measurement with 300 µL (150 µL each basal and apical) of ice-cold 70% ethanol (10 min, -20 °C) after being washed twice with PBS. Afterwards, the cells were washed again, and 500 µL of PBS was added apically and basally for storage at 4 °C until staining.

The fixed cells were washed twice with PBS (200 µL apical and basal) and incubated with 250 µL 0.2% Triton-X-100 (Sigma-Aldrich, St. Louis, MO, USA) for 10 min at room temperature, followed by a 10-minute treatment of PBS with 2% goat serum (blocking solution) (Gibco, Carlsbad, CA, USA). For the staining of the Zonula occludens-1 protein (ZO-1), 100 µL of a 1:100 dilution of mouse anti-human ZO-1 antibody (Cat: 610967; BD Transduction Laboratories, BD Biosciences, Thermo Fisher Scientific) was added each apically and basally and incubated for 60 min at 37 °C. Afterwards, the cells were washed three times with blocking solution, followed by the addition of 100 µL of a 1:100 dilution of rabbit anti-mouse claudin-5 antibody (Prod# 34-1600, Lot# SC249265, Invitrogen, Thermo Fisher Scientific) each apically and basally and incubation for 60 min at 37 °C. Following another three washing steps with blocking solution, the 1:500 diluted secondary antibodies goat anti-rabbit AlexaFluor-488 and goat anti-mouse AlexaFluor-594 (Thermo Fisher Scientific) were added (100 µL apical and basal) and incubated at 4 °C overnight. Finally, the cells were washed once with blocking solution followed by Aqua dest., before being subsequently mounted with ProTaqs MountFluor (Cat.No. 401603095, quartett, Berlin, Germany). The visualisation of the stained cells was performed by confocal laser-scanning microscopy at the Service Unit Microscopy of the Veterinary Centre for Resistance Research (TZR) with an inverted Leica Stellaris 8 FALCON microscope equipped with 405 nm laser, white light laser (WLL, 440–790 nm), acousto-optical beam splitter, Power HyD detectors and controlled by LAS X (version 4.5.0.25531). Images were acquired with a HC PL APO 63x/1.30 glycerine immersion objective. Image pixel size was 72 nm, z-step size was 333 nm, and the pinhole was set to one airy unit. The following fluorescence settings were used: Alexa 488 (excitation 499 nm; emission 504–595 nm) and Alexa 594 (excitation 590 nm; emission 595–660 nm). Line sequential acquisition mode (with line average = 4) was used to avoid bleed-through of fluorescence signals. Images were further processed linearly for brightness and contrast in ImageJ/FIJI and are displayed as maximum intensity projections [52].

### Statistics

All data were analysed using GraphPad Prism Version 10.2.3 (GraphPad Software, Boston, Massachusetts USA; www.graphpad.com). Since the data failed the Shapiro-Wilk test for normality we performed the statistical significance analysis using the Kruskal-Wallis test, followed by Dunn´s post-hoc multiple comparisons. *P* values < 0.05 were considered statistically significant, with significance levels indicated as *p ≤ 0.05, **p ≤ 0.01, ***p ≤ 0.001, and ****p ≤ 0.0001.

## Results

### Cytotoxicity of *A. cryaerophilus* in HT-29/B6 and T84 cells

**Fig. 1:**
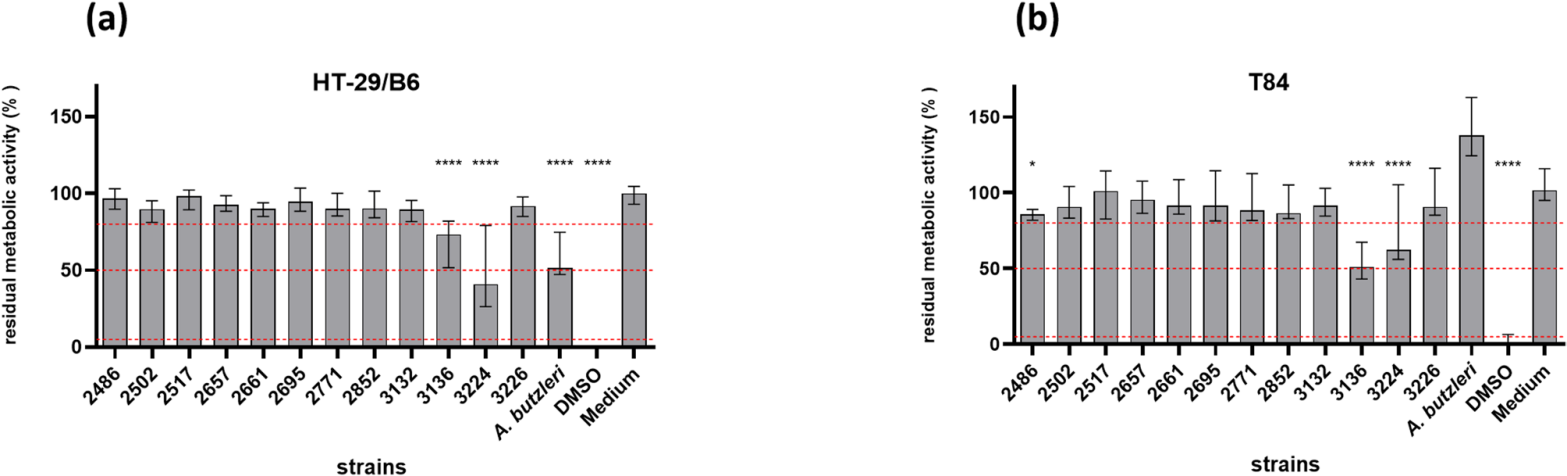
Induction of cytotoxicity in HT-29/B6 (**a**) and T84 (**b**) cells after infection with *A. cryaerophilus*. Induction of cytotoxicity after 48 h of infection with *A. cryaerophilus* (MOI = 1000) by measuring the metabolic activity with the WST-1 assay. Metabolic activity was normalised to the metabolic activity of uninfected cells (medium). DMSO and *A. butzleri* CCUG 30485 were included as controls. Three independent experiments with six replicates each were performed and are shown as medians with interquartile ranges; thresholds are displayed as red dotted lines and set for high cytotoxicity at 5%, moderate cytotoxicity at 50% and low cytotoxicity at 80% remaining relative metabolic activity; * *P* ≤ 0.05, **** *P* ≤ 0.0001 (Kruskal-Wallis test)

The impact of *A. cryaerophilus* on the metabolic activity of the HT-29/B6 and T84 cell lines was measured by the WST-1 assay. Impaired relative metabolic activity of human cells induced by bacterial infection was classified as low (51–80% remaining metabolic activity of the infected cells), moderate (5–50%), or high (less than 5%), as described by Brückner et al. (2020) [45]. DMSO treatment completely inhibited the metabolic activity of the tested cells (0.0% in HT-29/B6 and 1.0% in T84 remaining metabolic activity after 48 h of infection). HT-29/B6 cells showed compromised metabolic activity of more than 80% following infection by ten of the twelve tested *A. cryaerophilus* strains (Fig. 1a). Incubation with strain 3136 induced a low decrease in metabolic activity to 73.3% (*p* < 0.0001), and incubation with strain 3224 resulted in a moderate decrease to 41% (*p* < 0.0001). The reference strain *A. butzleri* decreased the metabolic activity to 51.6%, indicating a low level of cytotoxicity (*p* < 0.0001).

In T84 cells, *A. butzleri* did not significantly affect the metabolic activity of the cells in comparison to the control (Fig. 1b). In contrast, the impact of *A. cryaerophilus* strains 3224 and 3136 can be classified as low, with 62.5% and 51.0% residual metabolic activity, respectively (*p* < 0.0001). Strain 2486 lowered the metabolic activity but not below the threshold of 80%, resulting in 85.6% residual activity (*p* < 0.05).

When comparing the metabolic reduction induced by the respective *A. cryaerophilus* strains between both cell lines, no differences were observed (Table 2). The two strains that exhibited cytotoxicity in HT-29/B6 cells demonstrated a comparable effect in T84 cells, while the other strains did not reduce the metabolic activity of either cell line below 80%. In contrast, the *A. butzleri* strain only reduced the metabolic activity of the HT-29/B6 but not that of the T84 cells (*p* < 0.0001).

**Table 2:** Comparison of the cytotoxic effects of *A. cryaerophilus* on two different cell lines, HT29/B6 and T84

	HT-29/B6				T84				HT-29/B6 vs. T84
Strain	Median*			Quartile (lower/upper)	Median		Quartile (lower/upper)		*p* value ^a^
***A. cryaerophilus***									
2486	96.6		89.8 / 103.1		85.6		81.8 / 89.0		0.342
2502	89.7		81.1 / 95.3		90. 7		83.3 / 104.2		> 0.999
2517	98.3		89.4 / 102.3		101.1		82.7 / 114.4		> 0.999
2657	92.6		88.4 / 98.6		95.4		86.5 / 107.7		> 0.999
2661	90.1		85.1 / 94.0		91.3		85.9 / 108.7		> 0.999
2695	94.6		88.5 / 103.5		91.3		81.5 / 114.5		> 0.999
2771	90.0		85.3 / 100.2		88.4		81.7 / 112.7		> 0.999
2852	90.4		84.2 / 101.6		86. 7		83.0 / 105.2		> 0.999
3132	89.6		81.8 / 95.5		91.6		84.7 / 102.9		> 0.999
3136	73.3		51.8 / 82.1		51.0		43.1 / 67.4		> 0.999
3224	41.0		26.4 / 79.2		62.5		56.1 / 105.3		> 0.999
3226	91.8		85.1 / 97.8		90.7		85.2 / 116.2		> 0.999
***A. butzleri***									
CCUG 30485	51.6		47.3 / 74.8		138		124.5 / 163		**< 0.0001**

* Median and quartiles were calculated from at least three independent experiments with six replicates each. Values were normalised to the metabolic activity of uninfected cells (medium)

^a^ Kruskal-Wallis test; statistically significant differences are highlighted with p values in bold

### Adhesion and invasion potential of *A. cryaerophilus *in human colonic cell lines HT-29/B6 and T84

The ability of *A. cryaerophilus* to adhere to and invade human colon cells lines was investigated using HT-29/B6 and T84.

**Fig. 2:**
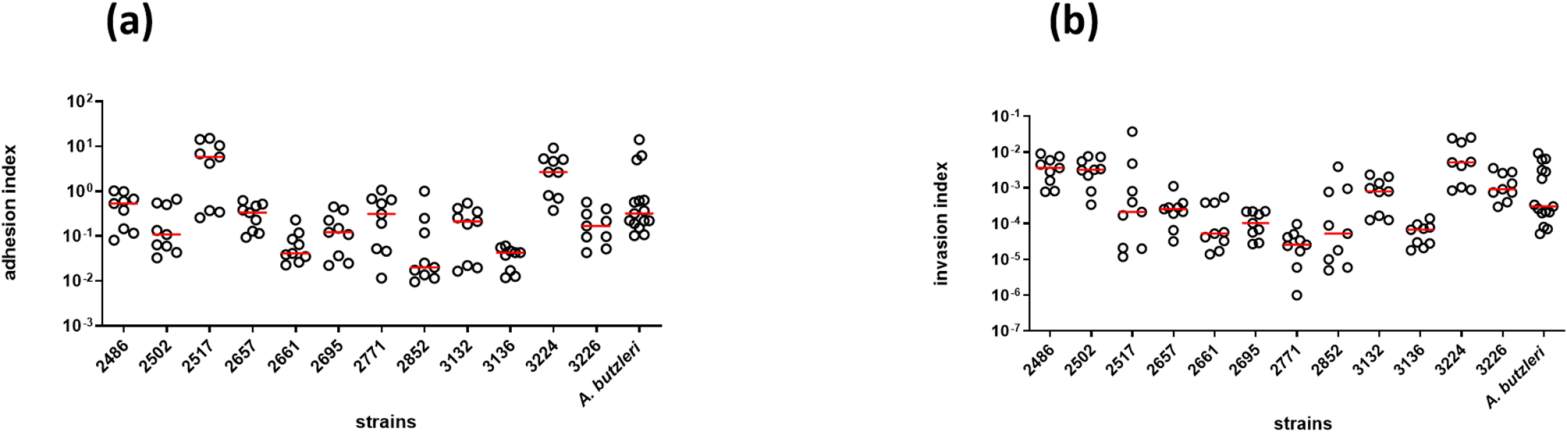
Adhesion (**a**) and invasion (**b**) capacity of twelve *A. cryaerophilus *strains in HT-29/B6 cells. Determination of the adhered bacteria was performed 1 h after infection (MOI = 100), and of the invaded bacteria 3 h after infection, followed by 2 h of gentamicin treatment. The indices were calculated as percentages of the inoculum (10^9^ CFU/mL). *A. butzleri* CCUG 30485 served as a positive control. Three independent experiments with three replicates each were performed and are shown together with the calculated medians

All twelve *A. cryaerophilus* strains successfully adhered to HT-29/B6 cells, with highest indices detected for strain 2517 (5.8 × 10^0^), followed by strains 3224 and 2486 (2.7 × 10^0^ and 5.3 × 10^− 1^, respectively) (Fig. 2a). The other strains exhibited adhesion indices ranging between 3.3 × 10^− 1^ (strain 2657) and 2.0 × 10^− 2^ (strain 2852).

The invasion ability was investigated following a gentamicin protection assay. The highest indices were observed for strains 3224 (5.2 × 10^− 3^) and 2486 (3.6 × 10^− 3^), whereas the lowest index was detected for strain 2771 (2.6 × 10^− 5^) (Fig. 2b).

The adhesion as well as the invasion indices of the control strain *A. butzleri* were found to be in the range of those of the tested *A. cryaerophilus* strains (3.2 × 10^− 1^ and 3.0 × 10^− 4^, respectively).

**Fig. 3:**
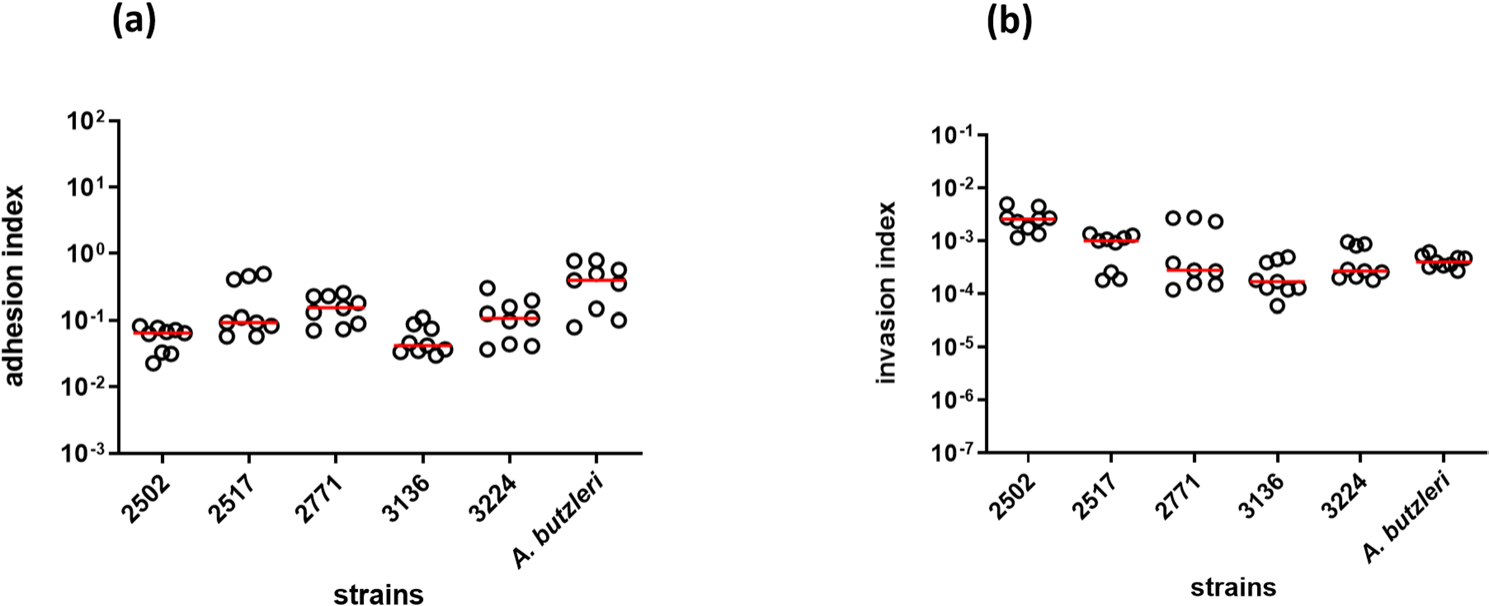
Adhesion (**a**) and invasion (**b**) capacity of five *A. cryaerophilus *strains in T84 cells. Indices were calculated compared with those of the inoculum (10^9^ CFU/mL), resulting in a MOI of 100, from three independent experiments with three replicates each. Single values with calculated medians are shown. *A. butzleri* CCUG 30485 served as a positive control

Two strains that were cytotoxic toward both cell lines (3136 and 3224) and three other strains displaying the whole range of invasion indices in HT-29/B6 cells (2502, 2517, and 2771) were chosen to further determine their adhesive and invasive potential on the T84 cell line (Fig. 3). The adhesion indices of all five *A. cryaerophilus* strains ranged between 4.2 × 10^− 2^ (strain 3136) and 1.5 × 10^− 1^ (strain 2771) in the T84 cells, which was slightly lower compared to *A. butzleri* (4.0 × 10^− 1^) (Fig. 3a). The invasion indices of all five strains in the T84 cell line were comparable with the index of *A. butzleri* (4.0 × 10^− 4^) (Fig. 3b). The highest indices were observed for strains 2502 (2.6 × 10^− 3^) and 2517 (1.0 × 10^− 3^), whereas for strains 2771, 3224 and 3136, lower indices (2.8 × 10^− 4^, 2.7 × 10^− 4^, and 1.7 × 10^− 4^, respectively) were detected compared with those of the *A. butzleri* strain.

When comparing the two cell lines, the two *A. cryaerophilus* strains 2502, and 3136, and the *A. butzleri* strain CCUG 30485 showed no significant difference in their adhesion and invasion capabilities (Table 3). However, for three strains differences between the two cell lines were observed. Strain 3224 exhibited higher adhesion and invasions indices in HT-29/B6 cells compared to T84 cells (*p* < 0.01 and *p* < 0.05, respectively). *A. cryaerophilus* strain 2517 exhibited only a higher adhesion index in the HT-29/B6 cell line (*p* < 0.05), while the invasion indices did not differ. Further, strain 2771 exhibited similar adhesion indices but a lower invasion index in HT-29/B6 cells compared to T84 cells (*p* < 0.05).

**Table 3:** Comparison of the adhesion and invasion capabilities of five *A. cryaerophilus* strains in HT-29/B6 and T84 cells

	HT-29/B6		T84		HT-29/B6 vs. T84
Strain	Median*	Quartile (lower/upper)	Median	Quartile (lower/upper)	*p* value ^a^
**Adhesion**					
***A. cryaerophilus***					
2502	1.1 × 10^− 1^	5.2 × 10^− 2^ / 5.3 × 10^− 1^	6.5 × 10^− 2^	3.2 × 10^− 2^ / 7.5 × 10^− 2^	0.6148
2517	5.8 × 10^0^	3.6 × 10^− 1^ / 1.2 × 10^1^	9.3 × 10^− 2^	7.0 × 10^− 2^ / 4.3 × 10^− 1^	**0.0272**
2771	3.1 × 10^− 1^	4.9 × 10^− 2^ / 6.6 × 10^− 1^	1.5 × 10^− 1^	8.2 × 10^− 2^ / 2.3 × 10^− 1^	> 0.9999
3136	4.3 × 10^− 2^	1.5 × 10^− 2^ / 5.1 × 10^− 2^	4.2 × 10^− 2^	3.4 × 10^− 2^ / 8.1 × 10^− 2^	> 0.9999
3224	2.7 × 10^0^	7.5 × 10^− 1^ / 5.2 × 10^0^	1.1 × 10^− 1^	4.3 × 10^− 2^ / 1.8 × 10^− 1^	**0.0020**
***A. butzleri***					
CCUG 30485	3.2 × 10^− 1^	1.9 × 10^− 1^ / 6.3 × 10^− 1^	4.0 × 10^− 1^	1.2 × 10^− 1^ / 6.7 × 10^− 1^	> 0.999
**Invasion**					
***A. cryaerophilus***					
2502	3.2 × 10^− 3^	1.5 × 10^− 3^ / 6.4 × 10^− 3^	2.6 × 10^− 3^	1.6 × 10^− 3^ / 3.6 × 10^− 3^	> 0.9999
2517	2.1 × 10^− 4^	2.1 × 10^− 5^ / 2.8 × 10^− 3^	1.0 × 10^− 3^	2.3 × 10^− 4^ / 1.2 × 10^− 3^	> 0.9999
2771	2.6 × 10^− 5^	1.2 × 10^− 5^ / 4.7 × 10^− 5^	2.8 × 10^− 4^	1.6 × 10^− 4^ / 2.5 × 10^− 3^	**0.0159**
3136	6.8 × 10^− 5^	2.5 × 10^− 5^ / 8.7 × 10^− 5^	1.7 × 10^− 4^	1.3 × 10^− 4^ / 4.2 × 10^− 4^	0.8744
3224	5.2 × 10^− 3^	9.6 × 10^− 4^ / 2.2 × 10^− 2^	2.7 × 10^− 4^	2.1 × 10^− 4^ / 8.5 × 10^− 4^	**0.0336**
***A. butzleri***					
CCUG 30485	3.0 × 10^− 4^	2.0 × 10^− 4^ / 3.2 × 10^− 3^	4.0 × 10^− 4^	3.3 × 10^− 4^ / 5.1 × 10^− 4^	> 0.9999

* Median and quartiles were determined from at least three independent experiments, each performed in triplicate. Indices were calculated compared to the inoculum (10^9^ CFU/mL)

^a^ Kruskal-Wallis test; statistically significant differences are highlighted with *p* values in bold

### Effect on epithelial barrier function

#### Transepithelial electrical resistance

The ability of *A. cryaerophilus* to affect the epithelial barrier function was evaluated by measuring the TER in T84 before and after infection. Herein, a high resistance is a sign for an intact barrier function, whereas a decrease in the TER hints at a disruption of the epithelial barrier, which subsequently can contribute to increased permeability against different ions, water or even larger molecules.

**Fig. 4:**
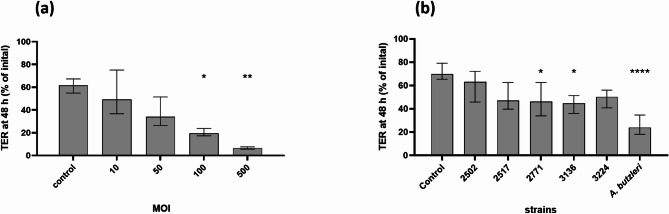
Transepithelial electrical resistance of T84 cells after infection with *A. cryaerophilus*. Transepithelial electrical resistance (TER) of T84 cell monolayers 48 h after infection with different MOIs (10, 50, 100, and 500) of *A. cryaerophilus* 3136 (**a**) and five strains of *A. cryaerophilus*, at a MOI of 100 (**b**). The measured resistance was normalised to the initial resistance before infection. Medium was included as a negative control, and *A. butzleri* CCUG 30485 was included as a positive control. Four replicates (**a**) and three independent experiments with three replicates each (**b**) were performed and are shown as medians with interquartile ranges. * *p* ≤ 0.05, ** *p* ≤ 0.01, **** *p* ≤ 0.0001 (Kruskal-Wallis test)

First, to determine the MOI for further investigations, T84 cells were infected with strain 3136 at MOIs of 10, 50, 100 and 500. After 48 h of infection, the initial TER was reduced to 49.3% by a MOI of 10, followed by a reduction to 34.1% by a MOI of 50 and to 19.5% by a MOI of 100 (*p* < 0.05), with the strongest reduction by a MOI of 500 to a residual TER of 6.6% (*p* < 0.01) (Fig. 4a). Based on these results, a MOI of 100 was chosen for subsequent assays.

The impact on the epithelial barrier function of five *A. cryaerophilus* strains (2502, 2517, 2771, 3136, and 3224) and *A. butzleri* strain CCUG 30845 was assessed. The *A. cryaerophilus* strains were chosen, as they included the two cytotoxic strains as well as three strains with varying invasion rates. Measurements after 48 h of infection revealed that two *A. cryaerophilus* strains, namely 2771 and 3136, as well as the *A. butzleri* strain, were able to significantly decrease the TER compared to the control, resulting in 46.1%, 44.5%, and 23.9% of initial TER, respectively (*p* < 0.05, *p* < 0.05, and *p* < 0.0001; Fig. 4b). The reduced TER induced by the three *A. cryaerophilus* strains 2502, 2517 and 3224 (63.0%, 47.0%, and 50.2%, respectively) was not significantly different compared to that of the control.

### Distribution of tight junction proteins

**Fig. 5:**
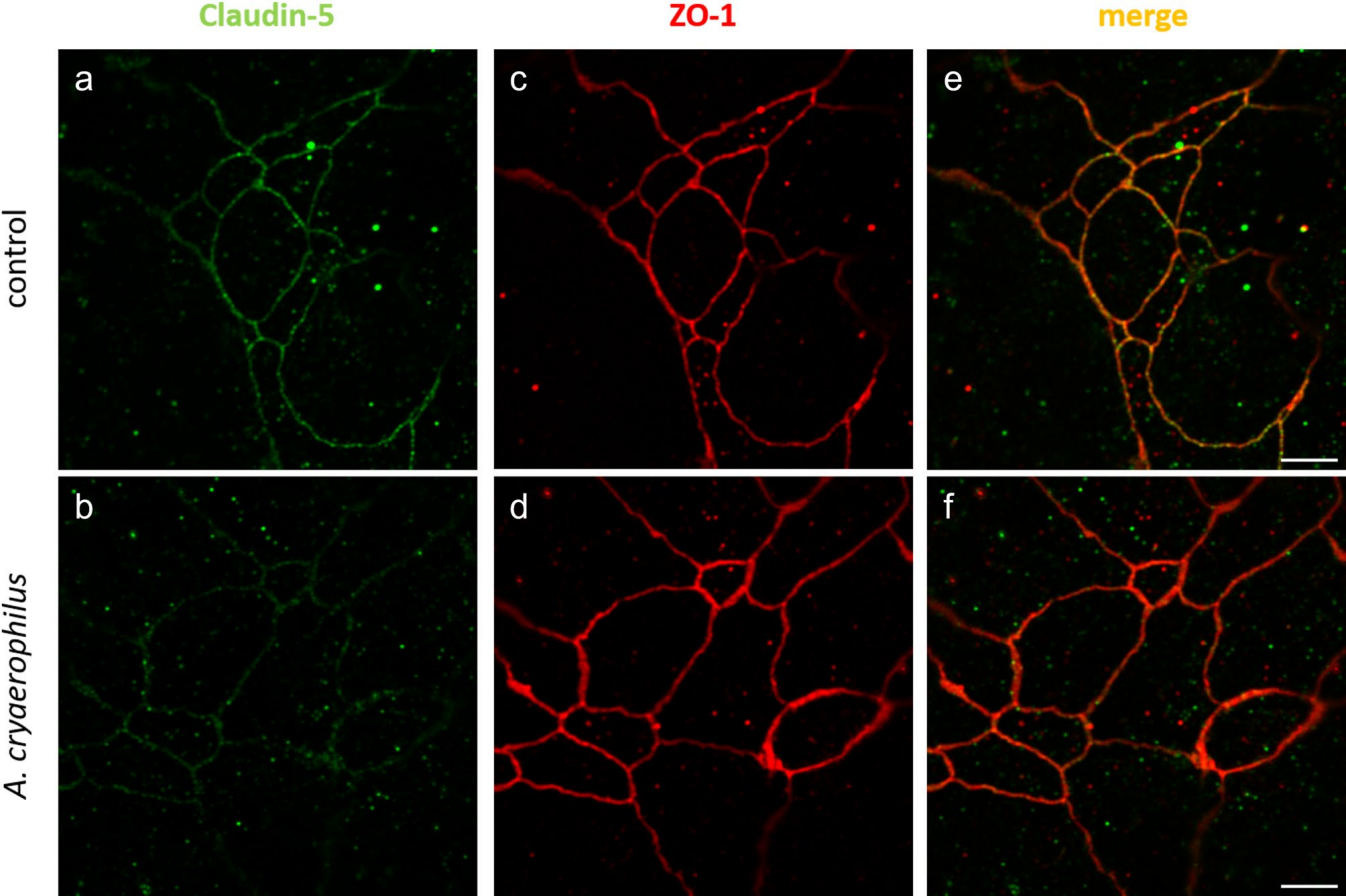
Immunofluorescence staining of tight junction proteins in T84 cells after infection with *A. cryaerophilus*. Immunofluorescence staining of tight junction proteins in the T84 monolayer 48 h after infection with *A. cryaerophilus* strain 3136. Tight junction proteins were stained with antibodies against claudin-5 (green, **a** and **b**) and ZO-1 (red, **c** and **d**), both of which were merged (yellow, **e** and **f**). Scale bar = 5 μm

To further investigate how *A. cryaerophilus* strain 3136 affects the epithelial barrier function, the tight junction proteins claudin-5 and ZO-1 of the T84 cell monolayer from the TER-assay were visualised by immunofluorescence staining (Fig. 5).

Confocal microscopy revealed a disruption of the claudin-5 signal in the cell membrane area of infected cells (5b) compared to the uninfected control cells (5a). The ZO-1 signal remained equally distributed in the tight junction domain in both infected and control cells (5d, 5c). Merged images revealed co-localisation of claudin-5 and ZO-1, indicated by a yellow signal, exclusively in uninfected cells (5e), whereas no co-localisation was detectable in infected cells (5f).

## Discussion

With relevance to health, the emerging foodborne pathogen *A. cryaerophilus* has been found to be the second most common *Arcobacter* species in human stool samples following *A. butzleri* [26]. Infections caused by *A. cryaerophilus* induce mainly prolonged watery diarrhoea [29, 47, 48]. Also in an animal model, *A. cryaerophilus* induced diarrhoeal disease in infected WISTAR rats [53]. However, knowledge regarding the pathogenic mechanisms of *A. cryaerophilus* is still scarce [44–46, 54, 55]. Nevertheless, as the few investigations regarding the adhesion and invasion rates of *A. cryaerophilus* have been performed on a single cell line, it is difficult to draw a general conclusion. Therefore, the aim of this study was to investigate the cytotoxicity as well as the adhesiveness and invasiveness on two further human intestinal cell lines, namely HT-29/B6 and T84. Both cell lines are able to form highly differentiated, polarised monolayers producing mucus, which is described to affect the ability of pathogens to colonize human intestinal cells [56–59]. Moreover, we evaluated how *A. cryaerophilus* affects the epithelial barrier function on T84 cell line. This cell line is characterised by its high degree of polarisation and high TER and is therefore particularly suitable for such investigations [60].

Currently, knowledge on the impact of *A. cryaerophilus* infections on the viability of human epithelial cells is still limited. Villarruel-López et al. (2003) observed morphological alterations in Vero cells following infection with *A. cryaerophilus*, such as elongation and vacuolisation, which could indicate cytotoxic effects [54]. Our results are pointing towards a strain-dependent cytotoxic effect of the investigated strains in both human cell lines. For two strains (3136 and 3224), we observed a low or moderate decrease in residual metabolic activity in HT-29/B6 cells and in T84 cells after infection. Our results are consistent with the findings of Brückner et al. (2020), who reported that approximately 20% of the included *A. cryaerophilus* strains cause a cytotoxic effect on the investigated cell lines [45]. The reason why only certain strains induce cytotoxicity remains unknown. The presence of cytotoxins that induce cell death by damaging nuclear DNA, such as the cytolethal distending toxin of *Campylobacter* spp. or *Helicobacter* spp., has not yet been identified in *A. cryaerophilus* strains [61–64]. Interestingly, for *A. cryaerophilus* the results of the cytotoxicity assay did not alter in comparison of the two tested cell lines, whereas this was the case for the *A. butzleri* strain CCUG 30845. This *A. butzleri* strain was able to reduce the metabolic activity to 51.6% in HT-29/B6 cells, whereas no reduction was detected in T84 cells. Differences between the cell lines might contribute to the observed differences in the cytotoxicity of the *A. butzleri* strain [65, 66]. For instance, variations of Toll-like receptor 4 (TLR-4), including its expression pattern and activation, are known to exist between the two cell lines [67]. Bacterial lipopolysaccharides or lipooligosaccharides (LPS or LOS) are ligands of this receptor, leading to the induction of inflammation and possibly resulting in cell death, and T84 cells are reported to respond less towards bacterial LPS than HT-29 cells do [67–69]. However, the role of TLR-4 activation by *Arcobacter* spp. remains controversial. Gölz et al. (2015) observed, that the *A. butzleri* strain CCUG 30485, which was also included in the present study, induced less colonic epithelial apoptosis and a weaker immunological response in secondary abiotic IL-10^−/−^ mice lacking TLR-4, compared to TLR-4-positive mice [70]. This suggests a potential involvement of TLR-4 in immunopathological processes and may contribute to the observed result that *A. butzleri* only induced cytotoxicity in HT-29/B6 but not in T84 cells. In contrast, Baztarrika et al. (2025) reported that infection of TLR-4-transfected HeLa57A cells with *A. butzleri* strains did not activate the TLR-4 receptor [71]. The differences in TLR-4 involvement between these studies might be due to strain-dependent differences in bacterial LOS/LPS structure or TLR-4 structures present in the different models used. Interestingly, Baztarrika et al. (2025) reported an activation of TLR-4 by *A. cryaerophilus*, which indicates that the LOS/LPS structure of some *A. cryaerophilus* strains could activate TLR-4 receptors and might induce cytotoxic effect thereby. However, despite the possible cytotoxic effect of *A. cryaerophilus* LOS/LPS, Johnson and Murano (2002) proposed the existence of a yet unidentified secreted toxin in *A. butzleri* and *A. cryaerophilus* as they observed morphological alterations in CHO, HeLa, and INT407 cells following infection with sonicated bacterial cultures and supernatants [64]. Such a toxin might explain a strain-dependent but TLR-4-independent cytotoxicity. Therefore, future research should investigate the cytotoxicity of supernatants from *Arcobacter* spp. to identify possible secreted toxins.

Moreover, invasion is known to potentially lead to cell death [72]. In our study, all considered *A. cryaerophilus* strains were able to adhere to as well as invade both cell lines investigated, with strain-dependent differences regarding the adhesion and invasion indices. In contrast, in the study by Ho et al. (2007) only two of the four strains included were invasive in Caco-2 cells, and only one was invasive in the porcine-derived cell line IPI-2I [55]. However, our findings are in line with other studies in which all included *A. cryaerophilus* strains were adhesive and invasive using the Caco-2 model [44–46]. Levican et al. (2013) reported adhesion capacities of *A. cryaerophilus* strains ranging from 5.37 to 6.31 log_10_ CFU/mL, and invasion levels between 2.31 and 3.06 log_10_ CFU/mL [46]. Based on the reported inoculum of 10^8^ CFU/mL and applying our calculation method, this corresponds to adhesion indices of approximately 2.3 × 10^− 1^ to 2.0 × 10^0^, and invasion indices ranging between 2.0 × 10^− 4^ and 1.2 × 10^− 3^, which are of similar magnitude to those observed in the present study. Similarly, Baztarrika et al. (2024) reported comparable adhesion capacities (corresponding to indices of approximately 1.9 × 10^− 1^ to 6.0 × 10^0^) but slightly higher invasion indices (2.8 × 10^− 3^ to 1.3 × 10^− 1^) [44]. Furthermore, similar indices could be calculated for well-described adhesive and invasive bacterial species, like *A. butzleri* and *Campylobacter* (*C*.) *jejuni.* On different human colonic cell lines indices for both species range from 10^− 4^ to 10^0^ for adhesion and from 10^− 5^ to 10^− 1^ in case of invasion [44, 46, 51, 73–75]. Differences of our results and those of previous studies investigating *A. cryaerophilus* could be due to varying protocols, strains, incubation times, cell lines, and calculation methods used.

Nevertheless, the majority of the investigated *A. cryaerophilus* strains exhibited adhesive and invasive capacities in the Caco-2, HT-29/B6 and T84 cell lines, suggesting that this is a general phenotype of this species. However, for some strains differences regarding the amount of adherent and invaded bacteria on different cell lines could be observed. While for two *A. cryaerophilus* strains and the *A. butzleri* strain no differences were determined, for strain 3224 both the adhesion and invasion indices were lower in T84 cells compared to HT-29/B6 cells. In contrast, strain 2517 had a lower adhesion index, and strain 2771 had a higher invasion index for T84 cells. As there was no general shift in the adhesion or invasion index between the two cell lines, we assume that both cell lines included in our study are suitable for investigating these phenotypes of *A. cryaerophilus* strains. Furthermore, our results suggest that bacterial factors themselves might be responsible for the variations in the levels of adherent and invasive bacteria in these cell lines. This is in line with the high genetic heterogeneity reported for *A. cryaerophilus*, which might be one cause of the different observed phenotypes [1, 76, 77]. Moreover, a correlation between invasion rate and cytotoxicity can be ruled out for the investigated *A. cryaerophilus* strains. Strains with the highest invasion rate did not exhibit cytotoxic effects, whereas both strains being significantly cytotoxic towards both cell lines showed a lower invasion rate compared to other strains. Similarly, in other pathogens, such as *Pseudomonas aeruginosa*, a lack of correlation between invasion and cytotoxicity has been confirmed [78].

As diarrhoea is the most frequently reported symptom of *A. cryaerophilus* infection in humans, an additional assay was conducted to assess the integrity of epithelial barrier function in the human colonic cell line T84 after infection with *A. cryaerophilus*. The epithelial barrier is maintained by epithelial cells and their paracellular proteins, such as tight junction proteins. These proteins are known to connect adjacent epithelial cells and are crucial for the regulation of paracellular transport and barrier function towards ions and solutes [79]. In addition to their barrier function, their fence function enables the polarisation of epithelial cells [80]. Damage to tight junction proteins can lead to a loss of cell-cell connections, resulting in a disruption of the integrity of the cell layer. A potential increase in permeability towards water and solutes can further contribute to leak-flux type of diarrhoea [81]. Moreover, a disturbed epithelial barrier can serve as an entry point for bacteria in the systemic circulation [82]. To the best of our knowledge, the impact of *Arcobacter* on the intestinal barrier integrity has only been investigated for *A. butzleri* [73, 83].

Therefore, the TER between the apical and basal sides of a T84 cell layer was evaluated after infection. The ability to reduce the TER of the T84 cell layer to 23.9% was observed for *A. butzleri* CCUG 30845, for which the ability to reduce the TER of a HT-29/B6 monolayer had already been demonstrated in earlier studies [73, 83]. Of the five included *A. cryaerophilus* strains, two reduced the initial TER to 44.5% and 46.1%. The observed reduction in TER suggests that infection with certain *A. cryaerophilus* strains contributes to the impairment of the epithelial barrier in T84 cells. Such disturbance of the intestinal barrier is recognised as a significant pathomechanism of diarrhoeal diseases caused by other similar bacteria, such as *Campylobacter* [84, 85]. Different mechanisms leading to barrier impairment have been described, including the invasion of pathogens, cytotoxicity and damage of tight junction proteins [83, 86]. However, invasion as a causative factor has not been demonstrated for the tested *A. cryaerophilus* strains, as all strains were able to invade T84 cells, but only certain strains caused a reduction in TER. Furthermore, no correlation was detected between the invasion rate and the TER decrease, as both strains that significantly reduced the TER exhibited lower invasion rates than most other strains did. The induction of cell death, as observed in the WST-1 cytotoxicity assay, may contribute to the dysfunction of the T84 cell barrier. However, only one of the two strains that exhibited cytotoxicity also reduced the TER significantly. Also, the *A. butzleri* control strain, which was not cytotoxic towards T84 cells, lowered the TER. Consequently, a direct correlation between TER reduction and cytotoxicity could not be consistently demonstrated, suggesting that additional mechanisms are responsible for the observed TER decrease.

Therefore, immunofluorescence staining of the tight junction domain was performed. The tight junction proteins claudin-5 and ZO-1 were stained and visualised by confocal microscopy.

While in the control monolayers, both tight junction proteins were properly co-localised in the tight junction domain with a yellow merge impression, we observed that infection with *A. cryaerophilus* strain 3136 led to a clear reduction in the immunofluorescence signal of claudin-5. At the same time, the cell borders themselves did not appear to be altered, as indicated by the unchanged ZO-1 signal in infected cells. This finding provides a molecular explanation for our functional TER measurement, in which the corresponding strain reduced the resistance to 44.5%. Furthermore, it indicates that *A. cryaerophilus* strain 3136 can impair epithelial barrier function and thereby may contribute to the gastrointestinal symptoms of watery diarrhoea (leak flux). It should be noted that other possibly affected tight junction proteins could have additional effects on the impairment of the epithelial barrier, but these were not analysed here. Other bacteria, such as *A. butzleri* and *C. jejuni*, are reported to affect various tight junction proteins, including claudin-1, claudin-5, and claudin-8, as well as occludin and E-cadherin [83, 87, 88]. Further studies are needed to obtain a more comprehensive understanding on the impact of *A. cryaerophilus* infections on the tight junction domain. Nevertheless, our observations align with those of Bücker et al. (2009), who reported a similar effect of *A. butzleri* on the structure of the tight junction domain in HT-29/B6 cells [83]. They showed that cells infected with *A. butzleri* exhibited lower protein expression levels of claudin-1, -5 and − 8. Additionally, a reduction of claudin-1 and − 5 and the redistribution of claudin-1 and − 8 into intracellular compartments were observed by confocal microscopy.

## Conclusions

The observed effects of the investigated *A. cryaerophilus* strains revealed a strain-dependent pathogenic phenotype in two human intestinal cell lines. All strains were able to adhere to and invade both cell lines, and two strains significantly decreased the metabolic activity of the infected cell lines. Additionally, the integrity of the epithelial barrier was compromised by two strains, as determined by a TER measurement, and a reduction of claudin-5 was shown for one strain by confocal microscopy. In conclusion, *A. cryaerophilus* is assumed to exhibit a potential risk to human health in a strain-dependent manner. 

## Data Availability

All the data generated or analysed during this study are included in this published article.
